# Methodological reflections on using pilot data from fracture patients to develop a qualitative study

**DOI:** 10.1186/1756-0500-4-508

**Published:** 2011-11-23

**Authors:** Renée Otmar, Mark A Kotowicz, Geoffrey C Nicholson, Julie A Pasco

**Affiliations:** 1 Department of Medicine, Northwest Academic Centre, Western Section, The University of Melbourne, Sunshine Hospital, 176 Furlong Road, St Albans Victoria 3021, Australia; 2 Department of Medicine, Barwon Health, PO Box 281, Geelong, Victoria 3220, Australia; 3 Rural Clinical School, School of Medicine, The University of Queensland, Private Mail Bag 2, Toowoomba, Queensland 4350, Australia; 4 Barwon Epidemiology and Biostatistics Unit, School of Medicine, Deakin University, PO Box 281, Geelong, Victoria 3220, Australia

## Abstract

**Background:**

Qualitative studies are particularly valued for their exploratory nature but, like other research methods, they do require careful planning to ensure rigorous study design. Our objective was to undertake a pilot study to inform the development of a larger qualitative study.

**Results:**

We conducted a series of brief interviews with out-patients in a hospital setting. The interviews were designed to elicit superficial information about whether (and how) post-fracture osteoporosis investigation and/or treatment were being initiated among patients receiving treatment or follow-up for a current or recent fracture. We used thematic analysis to identify key themes in the data that related to the broader research questions.

We analysed data obtained from 11 out of a total of 12 interviews conducted. Participants were male and female, aged 19-83 years of age (median age 57 years). Participants attended 2-8 medical appointments to seek treatment and follow up for a current or recent fracture. The following four overarching themes emerged from thematic analysis of the data: fracture event, referral pathway, osteoporosis investigation and/or treatment, and communication by health practitioners and staff.

**Conclusions:**

This pilot study was necessarily tentative and exploratory in nature, but provided a helpful snapshot of some typical experiences in the public health system following fracture. Several themes emerged for consideration in the design of the main study.

Despite its critics, theoretical sampling and saturation continue to provide sustainable methods for ensuring that relevant themes and categories are covered in sufficient depth and breadth, appropriate to the needs of the study.

## Background

We conducted a short pilot study whose aim was to inform the development of a larger qualitative study exploring barriers and enablers in the treatment and prevention of osteoporosis.

Worldwide, 0.83 per cent of the global burden of non-communicable disease can be attributed to fragility fracture[1,2]. Fragility fractures represent a significant public health problem in Australia, too; with direct costs alone representing public health expenditure of approximately $7 billion per annum[3]. Along with the human cost of fracture,[4] this burden is expected to increase considerably as Australia's population ages[5].

There is much evidence in the literature to support the use of pharmacological interventions to treat osteoporosis [6] and reduce the risk of fracture. Certainly, the number and types of medications available to treat osteoporosis have expanded in recent years. Good results in clinical trials, unfortunately, are not always reflected in everyday practice. Treatment can only be as good as those who investigate and prescribe it, and those who comply, adhere and persist with their prescribed treatments. It has been estimated that 80-90 per cent of those at very high risk of fracture remain undetected and untreated. This suggests that the health practitioners attending to these people are not investigating for osteoporosis and/or not initiating treatment. Furthermore, when medical practitioners do attempt to initiate treatment, many people are choosing not to accept it. Among those who do start treatment, a great many - perhaps as high as 68 per cent - do not continue beyond 12 months[7,8].

The main study aims to explore barriers and enablers in osteoporosis treatment, by examining knowledge, beliefs, attitudes and cultural models of osteoporosis, as held by consumers and medical practitioners. Given its broad scope, we considered it prudent to conduct a pilot to inform development of the main study design. This paper provides an overview of the pilot study and reflects upon its usefulness, in terms of contributing towards methodological rigour, as well as in planning the study.

## Methods

A series of brief interviews was planned for the pilot. These interviews, which were to be of 5-10 minutes' duration, were to be exploratory in nature. They were designed to elicit superficial information about whether (and how) post-fracture osteoporosis investigation and/or treatment were being initiated while patients were receiving treatment or follow-up for a current or recent fracture.

### Setting and participants

The study is being undertaken in the regional city of Geelong in south-eastern Australia. Geelong is part of the Barwon Statistical Division, which has a relatively stable population of 220,000[9]. The region's social, cultural and geographical demographics closely resemble national socio-economic indicators, making it an ideal setting for epidemiological and other research[10].

Participants for the pilot study were recruited from the Geelong Hospital's fracture clinic over two afternoons in May 2009, one week apart. The Geelong Hospital offers the only emergency department in the region and is part of Barwon Health, the single-largest health services provider in the region. The fracture clinic is run weekly within the Outpatients department of the Hospital, attending some 75-90 patients each Thursday for fracture treatment, assessment or follow up.

Criteria for inclusion in the pilot were age 18 years and older and being in receipt of treatment or follow-up at the fracture clinic for a current or recent fracture. Although we considered interviews with approximately 10 participants and equal numbers of men and women across the adult age spectrum would likely provide sufficient data for our purposes, we deliberately did not specify the number of interviews to be held, given the highly exploratory nature of the pilot study. We used a 'sequential sampling' method of recruitment, whereby the researcher selects sample cases until the amount of new information or diversity of cases is filled, so that a saturation point is reached [11]. We aimed to recruit roughly the same number of men and women, across the age spectrum from 18 years of age, and a reasonably wide range of fracture sites and treatment experiences.

### Conduct and ethics

The study was conducted in accordance with the World Medical Association Declaration of Helsinki [12] and approved by the Barwon Health Human Research Ethics Committee. All participants signed a plain language statement, consenting to their involvement in the study as well as presentation of the data at scientific meetings and publication in scientific/medical journals.

The plain language statement was handed to adult patients (those aged 18 years and over) upon arrival by the Outpatients' receptionists, fracture clinic nurses or research staff. In addition, prior to being interviewed all participants read (or had read to them) the plain language statement. Participants were deemed to have consented when they agreed to be interviewed and presented at the research interview room.

The interviews were conducted in a consulting room adjacent to the fracture treatment rooms. Signs were posted on the walls and doors of the clinic, advising that the research was taking place and directing people to the research interview room.

### Interview schedule

The Interview Schedule was designed to facilitate the recording (by hand) of participants' responses, including: interview date, participant's sex, age, fracture site, approximate date of fracture, whether or not the person had attended the hospital's Emergency Department and/or was admitted to hospital, the number of medical visits related to the fracture, whether or not the person had visited his or her GP/family doctor and free notes about the person's fracture event and referral pathway for treatment of the fracture.

### Analysis

Six men and six women participated in the brief interviews. One of the men interviewed was not sure that he had sustained a fracture to his wrist/thumb, and thus data from this interview was not included in the analysis. We used thematic analysis to interpret the data collected from the remaining 11 brief interviews with fracture patients, which included field notes.

The thematic analysis examined the free text that emerged in the course of the interviews and field notes. Thematic analysis can be used to explore patterns and trends in the data, both at the individual interview level and at the collective level[13]. This method provided the perfect means by which to quickly and effectively exploit the data for its secondary purpose - that of informing the next stage of the study.

For the thematic analysis, the first author (RO) coded the data manually, using open coding, and then identified themes emerging through axial coding, a subsequent step designed to make connections (or links) between codes in order to categorise aspects of the data[13]. These tentative themes would be confirmed (or not) in the course of the analysis. Given that the interviews were intentionally brief, the data available for analysis was limited and thus it was relatively straightforward to confirm the themes from the data, for their secondary purpose.

## Results

Subject characteristics were summarised using Minitab statistical software, release 15 (Minitab Inc., State College, PA, USA). Participants (n = 11) were aged 19-83 years, with a median age of 57 years (Table 1). The following results were obtained through the analysis of data collected from the five males and six females who completed the brief interviews.

**Table 1:** Mean age and fracture location, by gender

	All	Male	Female
**Participants**	11	5	6
**Median age (IQR)**	57.0 years (43.0-65.0)	43.0 years (21.0-56.5)	61.5 years (54.5-81.5)
**Distal forearm**	5	2	3
**Ankle**	3	2	1
**Scapula**	1	-	1
**Humerus**	1	-	1
**Upper leg**	1	1	-
**Patella**	1	1	-
**Foot**	1	1	-

As shown in Table 1 overall the women attending the fracture clinic were older than the men, and the fracture sites in women were limited to the distal forearm, upper arm, shoulder and ankle, whereas the men experienced fracture at all sites listed, except for the upper arm and shoulder.

### Fracture site

Most participants (5) had sustained a wrist fracture; other fracture sites were shoulder/upper arm (2), ankle (1), ankle and heel (1), ankle and upper leg (1) and kneecap (1). Most fractures were sustained within a period of one week to 8 weeks prior to interview; one participant was attending for follow up of a fracture sustained 8-9 months prior to interview.

### Number of medical visits

Participants each used between two and eight visits to seek treatment/follow up for their fracture.

### Key themes emerging from the data

The following four overarching themes emerged from thematic analysis of the data: fracture event, referral pathway, osteoporosis investigation and/or treatment, and communication by health practitioners and staff.

### Fracture event

As was to be expected, participants considered the ways in which they had sustained their fracture as important, and everyone was keen to share his or her story about how it had happened. For the purposes of this study, it is important to note that most (eight) participants reported having sustained a fracture as a result of a sporting, recreational or work-related accident, and that the fracture event had come as a surprise. It would be fair to say that participants felt embarrassed by having sustained the fracture, with several indicating that they considered it to have been both avoidable and a "nuisance":

"I felt so stupid!"

"What an idiot!"

The remaining three participants (all female) said they had sustained their fracture in the course of a low-trauma event, such as falling from standing height or less. Of these, the two older women (aged 65 years, wrist fracture; aged 83 years, upper-arm fracture) said that they had a previously received a diagnosis of osteoporosis and that their falls had been from standing height or less; the 65-year-old reporting having "tripped but nothing was there to trip over". The third woman (age 47 years) had sustained an ankle fracture while getting out of bed; this participant was the only one to express concern about the possible underlying cause of her fracture and the potential long-term consequences of having "weak bones".

### Referral pathway

Ten of the 11 participants included in this analysis had attended a hospital emergency department following their fracture. None had been admitted to hospital.

Three participants had been to see their family doctor prior to attending the hospital emergency department and/or fracture clinic. Of these, two had been referred to the hospital for further treatment, while the third, a 19-year-old male whose mother (accompanying him in the interview) described him as having a mild intellectual disability, had self-referred to the hospital emergency department after having had a plaster cast put on "too tight" at the family medical clinic.

Except for the 19-year-old described above, in general participants were satisfied that their medical visits were appropriate; none expressed a view that they had had too few or too many medical visits for treatment of the fracture.

### Osteoporosis investigation and/or treatment

Seven participants reported that they were not investigated for osteoporosis, nor had they had a discussion with their family doctor or other medical staff (for example, in the fracture clinic) about osteoporosis itself, or its investigation or treatment.

A 57-year-old woman who had broken her wrist while exercising at the gym reported that she had been told (by staff at the fracture clinic) that her "bone density is okay" and that she did not have osteoporosis. She had had an x-ray to confirm the fracture, but a bone mineral density scan was not performed.

The 47-year-old woman, reported above as having broken her ankle while getting out of bed, expressed concern that she had not been offered a test for bone mineral density:

"I thought it was a bit odd that me, a healthy, active person - I eat well and live a healthy lifestyle - should break my ankle just getting out of bed. I mean, okay, if I rolled or sprained it, but to break it, that worried me."

She had asked staff at the fracture clinic and her family doctor whether she needed to have a "bone density thing [scan]" but was told that it would be expensive because she did "not have osteoporosis".

As mentioned above, two women reported having a received a diagnosis of osteoporosis prior to fracture. The 83-year-old woman who had broken her upper arm during a fall at home reported that she had been taking medication for osteoporosis "for quite a while now", though she could not remember for how long. Although she recalled that she was taking a calcium supplement, when prompted she could not remember what else she was taking for osteoporosis:

"I am taking so many different pills - I have some other conditions, you know - that I can't recall what is for what. But I take all that I am told to take."

The 65-year-old woman who had broken her wrist "tripping" over something at home, possibly the carpet, reported that she had been taken the medication prescribed to treat her osteoporosis for some time, but had decided to discontinue therapy some time earlier. Upon prompting, she stated that she had been taking Fosamax (a bisphosphonate) "for about three years", but:

"... when all hell broke loose with that big women's study and the cancer [the Women's Health Initiative, United States] and I read about it, I decided to stop."

Asked if she had discussed this with her family doctor, she replied:

"Yes, I did, and he said it was up to me, so now I only take Caltrate [a calcium supplement] and magnesium."

### Communication by health practitioners and staff

Many participants expressed their dissatisfaction about aspects of their fracture investigation and treatment. Some complaints related directly to practical aspects of fracture treatment. One participant reported that he had been in severe pain and had become ill as a result of a plaster cast set too tightly on his leg and ankle at his regular family medical practice. Another participant reported that she had had to have her wrist "rebroken", which she interpreted as the plaster cast not having been "properly set" after the initial fracture.

Other complaints related to communication by health practitioners and staff. Participants felt that their treatment and/or rehabilitation were not adequately explained. Several participants said they did not understand why they had to have more than one x-ray, or why they had had to have a plaster cast removed and re-set, often more than once. They interpreted these measures as incompetence; that staff "don't know what they are doing".

As mentioned above, one participant said that she had expected (and requested) further investigation for osteoporosis; she also expressed disappointment that advice was not given to her in the fracture clinic regarding post-fracture rehabilitation of her ankle; instead, she had independently sought advice from a physiotherapist.

## Discussion

The most common fracture site among our participants was to the wrist, and this reflects fracture prevalence rates in the Australian community, where distal forearm fractures have been reported as the third most common among both men and women over the age of 35 years[14]. Given the relatively young age profile of participants, it is not surprising that most fractures occurred in the course of sporting, work or other strenuous activity.

That participants were often embarrassed by the fact that they had sustained a fracture must be of interest to our main study - it would be important for the study to explore whether this reflects a general attitude in the community. Our future study might also explore why a fracture might be embarrassing.

Although participants had the story of how they sustained their fracture at top of mind, many were preoccupied with the event itself, rather than seeking deeper explanations for their fracture. Indeed, many did not appear to be aware that there could be an association between the fracture and their bone health. For example, the 65-year-old woman who tripped and fell from standing height but could not explain what she had tripped on did not appear to make a connection between the fall and the fact that she had an existing diagnosis of osteoporosis. However, this was a brief interview, and limited to discussion and reporting at a superficial level. Therefore, the main study would do well to investigate in greater depth people's knowledge, beliefs and attitudes in relation to both fracture and osteoporosis, and associations between the two. Topics for discussion might include definitions and understandings of osteoporosis (what is it and what causes it?), as well as an exploration of possible risks of having osteoporosis.

Certain fracture patients - perhaps those better informed about osteoporosis or health issues in general - may be alerted in the context of sustaining their fracture to seek advice or further investigation for osteoporosis. One example is the 47-year-old who broke her ankle while getting out of bed. She had a strong sense that "something was not right" about the fact that she had sustained a fracture, given her age and perceived health status, and had expectations about how this should be considered by those providing medical care for her fracture. Again, the main study would do well to investigate people's expectations of their health carers. Are there differences in expectations between the sexes - and/or age groups? Why are some people more proactive in their health-seeking behaviours than others? Given that the 47-year-old woman was the only one of the three women (and also the younger of these) who had sustained a low-trauma fracture who questioned the medical care she was receiving, in addressing the research questions the study might consider generational differences in expectations of, as well as interactions with, the health system.

It should be noted that, given the aetiology of osteoporosis, it may not have been relevant for most participants to have had a discussion about osteoporosis with their treating medical practitioner.

A topic of acute interest to the main study is patient adherence to osteoporosis treatment. Two participants reported having been prescribed treatment for osteoporosis. One of these, an older woman, reported being adherent to her prescribed medication but, alarmingly, she did not appear to understand which medications were to be taken for which conditions. It should also be noted here that the report on the Women's Health Initiative [15] study referred to by the other participant was in fact related to hormone therapy (HT) and possible association with increased risk for breast cancer, rather than the bisphosphonates she was prescribed for osteoporosis. Clearly, patients' perspectives on treatment adherence and their understanding of their treatment and how it works will be high on the main study's priority areas for investigation.

Attendance at a hospital emergency department tends to be the first port of call for people who sustain a fracture, suggesting that subsequent attendance at the fracture clinic presents an ideal - in some cases, the only - opportunity for initiation of osteoporosis investigation, where warranted. It may be helpful in our study to look at if/when and how initiation of a discussion about osteoporosis and/or investigation takes place in the course of fracture treatment in the hospital setting. We would be interested in exploring barriers and enablers to such investigation. Such research might explore practitioners' perspectives of osteoporosis investigation in the hospital setting and compare initiation of osteoporosis investigation in the acute setting of the hospital fracture clinic with community settings such as family medical clinics.

Analysis of the brief interviews that made up this pilot study has highlighted gaps in communication between healthcare staff and patients. That some participants did not understand why they had to have more than one x-ray is an example. Another was patients' lack of understanding regarding their prescribed pharmaceutical treatment. The main study's aims are to investigate barriers and enablers to fracture treatment and prevention by exploring, among others, people's preferences for receiving health information about osteoporosis, including initiation of discussion, investigation, diagnosis and treatment by their health practitioner (including their preferred health practitioner for osteoporosis investigation and treatment). It is hoped that the study's findings will provide recommendations for bridging that critical gap in communication.

## Methodological reflections

The key factor influencing this pilot was its purpose to inform the design of the main study, which aims to explore barriers and enablers in fracture treatment using a qualitative study design. In retrospect, we could have achieved the same findings with fewer interviews - most notably those with younger participants. There were strong similarities in the data obtained from the older participants (45 years and older), and the data obtained from the younger men, in particular, did not advance the aims of the main study. Qualitative studies often are criticised for a perceived weakness in not having firmly established, ideal sample sizes, and for their reliance upon the so-called "vague" concept of saturation[16]. However, in this pilot study, gathering more data from a bigger sample size did not necessarily help in furthering the study's aims. What would have been more helpful would have been to use a process by which data were collected and analysed "iteratively until saturation [was] achieved", [16] p. 20, rather than first collecting the data and then waiting analyse them all together. This is an important finding that we hope will guide researchers wanting to conduct a pilot study using qualitative methods - theoretical sampling and saturation remain highly valuable methods in this context.

Diversity in our sample of interviewees was likely limited by our recruitment in a hospital setting. However, given the nature of the pilot, it would have been difficult and time-consuming to recruit fracture patients from the 66 medical practices scattered across the Geelong region. The main study will increase the potential for diversity by recruiting participants from across the broader geographical region.

While the study was successful in that it achieved its primary purpose, its findings are necessarily limited to its specific local context, and these may not be generalisable to the broader Australian population or, indeed, other dissimilar populations.

## Conclusions

The brief interviews that made up this pilot study were necessarily tentative and exploratory in nature, but provided a helpful snapshot of some typical experiences in the public health system following fracture. Analysis of the data suggests several themes for consideration in designing the focus of consultations that will make up the basis of the main study. This provides the authors with a golden opportunity to develop the study design so that the approach and methods employed provide a solid and rigorous basis from which to explore the research questions.

Despite its critics, theoretical sampling and theoretical saturation continue to provide sustainable methods for ensuring that relevant themes and categories are covered in sufficient depth and breadth, appropriate to the needs of the study. As is continuously demonstrated in the increasing number of qualitative studies that are conducted across the world each year, one size cannot - and should not - fit all in qualitative methodology.

## Competing interests

The authors declare that they have no competing interests.

## Authors' contributions

All authors designed the study. RO conducted the interviews, and JAP assisted in recruiting participants for interview. RO analysed the data and drafted the manuscript. All authors read and revised the manuscript, and have approved the final manuscript.

## Authors' information

RO is currently completing a PhD using qualitative methodologies. She has a background in health communications and has previously conducted focus group studies on alcohol and marketing. MAK is an endocrinologist and an experienced clinical investigator. GCN is a research clinician with broad interests in metabolic disorders from clinical, epidemiological and laboratory perspectives. JAP is an epidemiologist with considerable experience in conducting observational studies focused on osteoporosis. The authors have mainly published in medical and health science journals.
